# Note from Prof. Rea

**Published:** 1867-12

**Authors:** R. L. Rea

**Affiliations:** 119 Clark Street


### *Note from Prof, Rea.*

WILL the physician from Iowa, one of the Alumni of Rush Medical College, who requested me to furnish him a proper person for partner, please send me his address again, as I mislaid it, and have one who will suit him.

R. L. REA, 119 Clark Street.

